# Acid Resistance of Glass Ionomer Cement Restorative Materials

**DOI:** 10.3390/bioengineering7040150

**Published:** 2020-11-22

**Authors:** Dinuki Perera, Sean C. H. Yu, Henry Zeng, Ian A. Meyers, Laurence J. Walsh

**Affiliations:** UQ Oral Health Centre, The University of Queensland School of Dentistry, 288 Herston Road, Herston, QLD 4006, Australia; d.perera@uq.net.au (D.P.); s.yu@uq.net.au (S.C.H.Y.); henry.zeng@uq.net.au (H.Z.); ian.meyers@uq.edu.au (I.A.M.)

**Keywords:** dental erosion, glass ionomer cement, biomimetic, demineralization, acidity, degradation

## Abstract

In view of the need for aesthetics, restorations of teeth will typically be completed using tooth colored restorative materials. With the advent of biomimetic restorative materials, such as glass ionomer cements (GIC), much greater emphasis is now being placed on how well such materials can resist the challenge of acids that are present in foods and drinks, or gastric contents that are regurgitated. This laboratory study compared the dissolution and behavior of five GIC materials (GC Fuji^®^ VII, GC Fuji^®^ Bulk, GC Fuji^®^ IX Fast, Fuji^®^ IX Extra and GC Equia^®^ Forte Fil) when exposed to three acids (citric acid, phosphoric acid and lactic acid), versus ultrapure deionized water, which was used as a control. Discs of each material GIC were submerged in solutions and percentage weight changes over time determined. Subsequently, the GIC materials were also placed as a part of standardized Class II sandwich restorations in bovine teeth (*n* = 20), and submerged in the solutions, and the extent of GIC dissolution and protection of the adjacent tooth was scored. Weight loss increased with time and with acid concentration. Overall, the most soluble material was GC Fuji^®^ IX Extra, while GC Fuji^®^ IX Fast and GC Fuji^®^ Bulk were less soluble, and the least soluble material was GC Equia^®^ Forte Fil. The most destructive solution for both the discs and for GIC restorations in teeth was 10% citric acid, while the least destructive acid was 0.1% lactic acid. The more recent GIC materials GC Fuji^®^ Bulk and GC Equia^®^ Forte Fil showed increased acid resistance over the older GIC materials, and this further justifies their use in open sandwich Class II restorations in more hostile environments.

## 1. Introduction

Tooth colored restorative materials used in conventional dental practice comprise those which are chemically inert (such as ceramics, hybrid ceramics, and resin composites (RC)), and those which ionically interact with the oral environment, such as glass ionomer cements (GIC).

The survival of restorations in the oral cavity of older patients is one of the greatest challenges facing modern dental practices. Many elderly patients have an adverse, highly acidic oral environment, which could be due to salivary gland side effects of prescribed or over-the-counter medicines, systemic disease (particularly diabetes mellitus and subclinical dehydration), or various forms of gastric reflux [1,2]. GIC restorations can act as a biomimetic dentine replacement, having similar thermal expansion properties to natural tooth structures, or as a bulk restorative for use in sites with low compressive loads [3]. Their common applications are for restoring carious lesions located on root surfaces, or interdentally (using the sandwich technique, where they are overlaid with a resin composite material). For interdental restorations, the “open” sandwich technique has been advocated to decrease the incidence of marginal caries due to the ability of GIC restorations to act as a “sacrificial anode”, buffering acid in the process, and protecting the adjacent tooth structure while undergoing gradual dissolution after prolonged acid exposure [4,5].

To try to improve the caries preventive and tooth protective effects of GIC, a range of modifications have been made, including altering the glass particles, including strontium, and adding in casein phosphopeptide amorphous calcium phosphate CPP-ACP [6,7]. The strontium can assist in remineralization, substituting for calcium ions [8]. An important issue with older types of GIC, such as GC Fuji^®^ IX Fast (F9F), GC Fuji^®^ IX Extra (F9X) and GC Fuji^®^ VII (F7) is that they are relatively prone to dissolve when exposed to prolonged low pH conditions. One way of demonstrating this is to expose standardized samples of GIC materials to calcium chelators such as citric acid, and then assess the change in mass which occurs over time, as the samples are exposed to this erosive challenge. Such an approach is well established as a means for testing the acid resistance of GIC [9,10,11]. The present study used this approach to assess the acid solubility of new GIC materials compared with older GIC materials, and also assessed their behavior in sandwich restorations kept in acidic solutions. According to the manufacturer, these new high-viscosity GICs, namely GC Fuji^®^ Bulk (FB) and GC Equia^®^ Forte Fil (EFF), claim to have improved physical properties and faster setting times because of ultra-fine glass particles embedded in a stronger matrix of higher molecular weight polyacrylic acid [12,13]. Such materials could also have increased resistance to acid dissolution. To represent typical acid challenges, in the present study samples were exposed to lactic acid (produced by cariogenic dental plaque biofilms), citric acid (present in citrus-based juices and carbonated beverages), and phosphoric acid (present in cola drinks), following methods used in previous studies [14,15].

## 2. Materials and Methods

A pilot study was undertaken to assess the influence of acid concentration and time, tracking discs of material over 14 days to assess their behavior in the three test acids at different concentrations (e.g., for citric acid, 0.1, 1 and 10%). Since weight loss was found to be concentration-related, based on the findings from this pilot study, the final concentrations selected for the main study with discus and with restorations in teeth were 0.1% lactic acid, 10% citric acid, and 0.2% phosphoric acid, as these gave notable effects at 7 days and were also concentrations that had been used in previous studies, thus providing a valid comparison to previous work. As a point of reference, lemon juice contains approximately 4.8% citric acid, whereas cola drinks contain up to 0.07% phosphoric acid [16,17].

To assess the dissolution of discs of various GIC materials, a matrix design was used, with standardized discs each of five GIC materials (F7, F9F, F9X, FB, and EFF) (GC Corporation, Tokyo, Japan) exposed to deionized water of the three acids over seven days, with *n* = 6 samples in each group, following a repeated measured design where all samples were compared to their situation at baseline. All acid solutions were prepared using reagent grade acids. To prepare discs of materials, a thin layer of petroleum jelly was used as a separator and was applied to the internal surface of stainless steel washers, providing molds of 5 mm diameter and 1 mm height. Six discs of each GIC were prepared. Materials were mixed following the manufacturer’s instructions using a programmed mixer, and loaded into the washers, and a glass slide applied during setting. After removing the discs from the washers, a coarse grade 3M Soflex™ disc was used to remove excess flash. The baseline weight was measured using a microbalance (accuracy 0.1 mg) and each sample was photographed against a CMYK color reference standard using a microscope camera at 10× magnification.

Each disc was placed into the flat-bottomed well of a 24-well cell culture plate, and covered with 2.0 mL of the appropriate solution. The solution was replenished each day. The discs were removed and dried after three days and again after seven days, and their weight recorded after blotting the discs dry. Photographs were taken at each time point. The percentage weight change for each disc was calculated, and then group data were collated. Separate statistical analyses were conducted for the effect of solution, the effect of material, and the effect of time, using Instat version 3.1 software (GradphPad, San Diego, CA, USA). All data sets were assessed for normality using the Kolmogorov–Smirnov test. For combinations of acid and time, and material and time where all groups passed the normality test, a one-way ANOVA with Tukey post-hoc analysis was performed to identify significant differences. For combinations where not all groups passed the normality test, a Kruskal–Wallis test with Dunn’s post-hoc analysis was performed.

In the second part of the study, the extent of dissolution of GIC materials in open sandwich Class II restorations when exposed to various acids was assessed using the same acid challenges, with a semi-quantitative grading system. Extracted single root anterior bovine teeth (*n* = 24) of similar size and shape were collected with the approval of the University of Queensland institutional animal ethics committee, approval number ANRFA/DENT/523/17) from a commercial abattoir. All remnants of the periodontal ligament were removed with periodontal curettes. To ensure a consistent volume of the various restorative materials was used in each tooth, standardized cavity preparations were made, using individual 20 mm × 20 mm × 20 mm polyvinylsiloxane putty keys to locate the root of each tooth. Four standardized 1.5 mm × 2.0 mm × 5.0 mm occluso-lateral cavities (SD ± 0.2 mm) were prepared in each tooth using a cylindrical diamond bur in an air turbine handpiece, ensuring that dentin remained on the cavity floor. The dentin in each cavity was conditioned with 10% polyacrylic acid (GC Corp, Tokyo, Japan) and restored with a 3 mm thick three GIC base, using a different GIC material (F7, F9F, FB, EFF) for each cavity within the same tooth. Materials were mixed and handled according to the manufacturer’s instructions. After 24 h, the GIC bases and cavity walls were etched with 37% phosphoric acid, then a bonding agent (Scotchbond™ Universal Adhesive, 3M Oral Care Solutions Division 3M Center, St. Paul, MN, USA) was applied, and 2 mm of a resin composite (shade P-A2 G-aenial™, GC Corporation, Tokyo, Japan) placed on the occlusal aspect, leaving a 2 mm portion of the GIC base exposed as an open sandwich. The restorations were polished using 3M Soflex™ discs. In total, six open sandwich restorations of each GIC material were prepared for each solution, with a total of 96 open sandwich restorations.

Two photographs at 10× and 20× magnifications were taken of each restoration using standardized lighting and magnification. The teeth were then removed from their individual putty keys and placed into 12 well culture plates (one per well), and covered with 3.0 mL of the appropriate acid or with deionized water. After 7 days, the teeth were removed, dried by blotting, and two photographs were taken of each restoration. All teeth were examined using LED lights and 2.5× magnifying loupes, and periodontal probes were used. Scores were assigned by three independent examiners using the following criteria: (1) GIC surface loss: 0 = No axial surface loss of GIC; 1 = axial surface loss <0.5 mm; 2 = axial surface loss >0.5 mm; (2) GIC color change: 0 = no color change; 1 = color change observed; (3) GIC surface irregularity: 0 = no surface irregularity; 1 = surface irregularity (such as pitting or roughness); (4) Resin composite material interface with the GIC: 0 = no changes to adjacent RC restoration; 1 = change to adjacent RC restoration observed; (5) Changes to the dentin bordering the GIC: 0 = No changes to adjacent dentin; 1 = visual changes to adjacent dentine. Component and aggregate scores were then tallied.

## 3. Results

**Figure 1:**
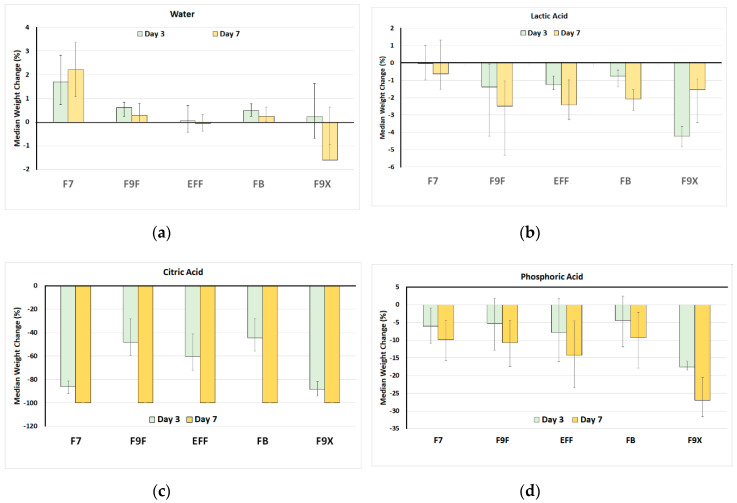
Median percent weight change of glass ionomer cements (GIC) discs after exposure to (**a**) water, (**b**) 0.1% lactic acid, (**c**) 10% citric acid, and (**d**) 0.2% phosphoric acid. In each series the sequence of GIC materials is GC Fuji^®^ VII (F7), GC Fuji^®^ IX Fast (F9F), GC Equia^®^ Forte Fil (EFF), GC Fuji^®^ Bulk (FB) and GC Fuji^®^ IX Extra (F9X). Error bars show 95% confidence intervals.

For discs of GIC material, an initial weight change was anticipated from baseline to day 3 because of sorption of water by some materials (Figure 1). In deionized water, at day 3 the highest initial weight gain occurred with F7. This was maintained at day 7, remaining above baseline. At 10 days, F9X lost weight, being the only material to do so.

For the three acids, overall, the greatest effects were seen with citric acid, followed by phosphoric acid, and then by lactic acid. The most acid soluble materials in citric acid were F7 and F9X, both experiencing around 90% weight loss after 3 days, while other GIC materials had lost 40–60% at the same time. All five GIC materials were completed dissolved away at day 7 in citric acid.

For phosphoric acid, the most soluble material at both 3 and 7 days was F9X, while all other materials showed similar performance, with a doubling of weight loss from around 5% at day 3 to around 10% at day 7.

For lactic acid, once again the most soluble material at both 3 and 7 days was F9X, while F9F, EFF, FB showed a similar performance, with a doubling of weight loss from around 1% at day 3 to around 2% at day 7. F7 did not show any significant change in weight when exposed to phosphoric acid.

Comparing the effect of the solution, for one material at one timepoint, there was a consistent ranking for weight loss from citric acid (the most, to phosphoric acid, to lactic acid (the least)). For many combinations of material and time, the lactic acid effects were so small that they did not reaching the threshold for statistical significance.

Overall, the least soluble materials were F9F, FB and EFF, with no significant differences between these.

**Figure 2:**
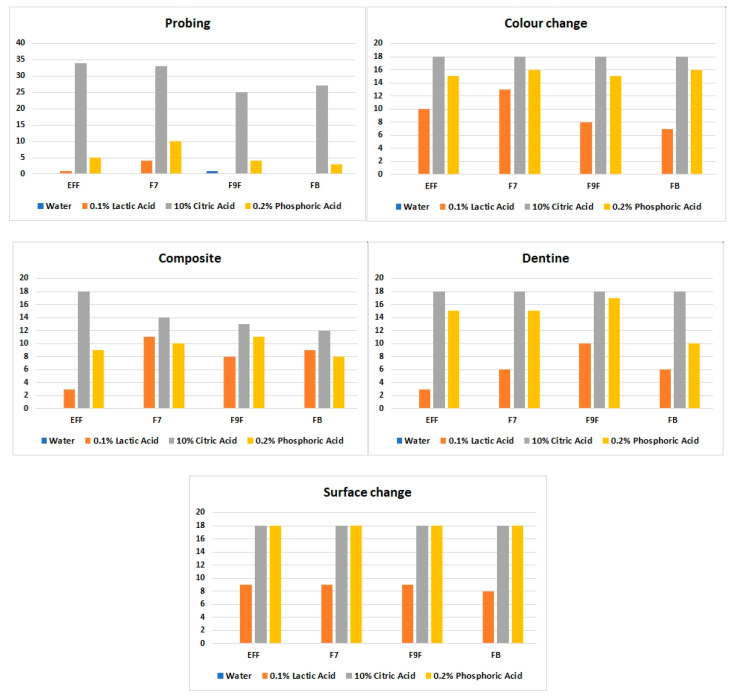
Cumulated assessor scores for qualitative assessments for open sandwich restorations on bovine teeth. From the top left, probing depth at the GIC surface, GIC color change, resin composite border changes, dentin border changes, and GIC surface irregularities. In each series the sequence of GIC materials is GC Equia^®^ Forte Fil (EFF), GC Fuji^®^ VII (F7), GC Fuji^®^ IX Fast (F9F), and GC Fuji^®^ Bulk (FB). Each sandwich restoration was assessed after seven days of media exposure (*n* = 24).

In the second part of the study, the solubility of GIC was assessed in the setting of the sandwich restoration. Immersion in water did not affect any of the parameters that were assessed. Once again, the most destructive acid was citric acid, followed by phosphoric acid and then by lactic acid. EFF and FB were the materials that were overall the least affected by exposure to acid, and F9F and F7 the most. Data for aggregated scores are shown in Figure 2.

**Figure 3:**
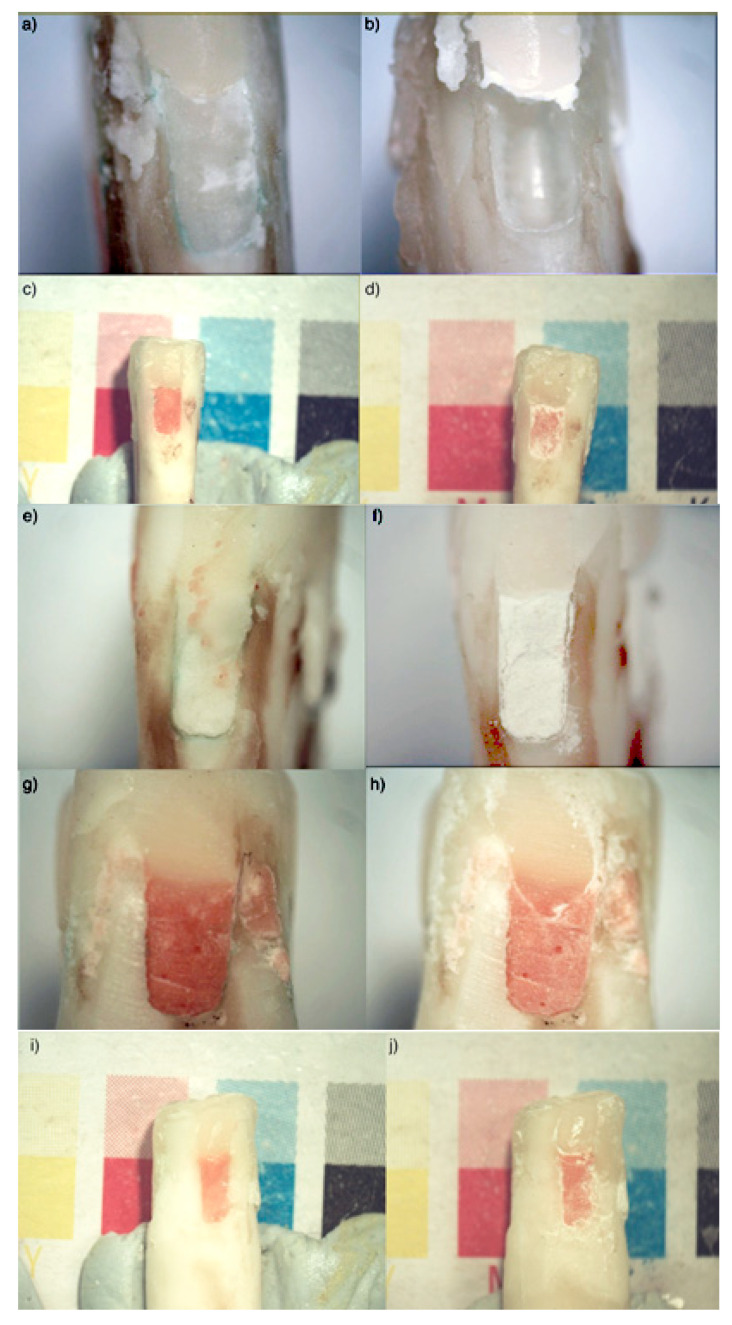
Open sandwich Class II restorations on bovine teeth, before (**left**) and after (**right**) 7 days of exposure. (**a**,**b**) GC Equia^®^ Forte Fil (EFF) with citric acid, showing considerable surface loss. (**c**,**d**) GC Fuji^®^ VII (F7) with phosphoric acid. The color is lighter. (**e**,**f**) GC Equia^®^ Forte Fil (EFF) with phosphoric acid, showing surface irregularities in the form of pitting, cracking and roughness. (**g**,**h**) GC Fuji^®^ VII (F7) with lactic acid, showing increased whiteness at the restoration margin. (**i**,**j**) GC Fuji^®^ VII (F7) with phosphoric acid. The adjacent dentin border shows increased whiteness.

GIC color change was observed more in samples exposed to 0.2% phosphoric acid and 10% citric acid than in those exposed to 0.1% lactic acid (Figure 3). The most common color change was the material appearing whiter across time. GIC surface irregularities were most apparent after exposure to 10% citric acid and 0.2% phosphoric acid, across all materials, and included surface roughness and pitting. In terms of the interface between the GIC and the adjacent RC restoration, visual disintegration of the interface was seen, especially with citric acid. Changes to the dentin border presented as increase in whiteness, as well as increased softness of the adjacent dentine when tactile probing was undertaken.

Overall, the results of the bovine teeth portion of the study followed those for the discs. Citric acid was the most destructive medium with the highest cumulative score for all parameters, while FB was the least soluble material.

## 4. Discussion

This study provides several insights into the acid solubility of new generation viscous GIC materials versus older GIC materials, from the same manufacturer, with the newer materials, such as GC Equia^®^ Forte Fil and GC Fuji^®^ Bulk being less soluble than older materials, such as GC Fuji^®^ IX Extra and GC Fuji^®^ VII. The findings also indicate that assessing weight change after three days of exposure to 10% citric acid provides a straightforward screening approach, since at this timepoint any highly soluble materials will have dissolved into the acid, and this can be assessed visually even before the sample is weighed.

The reasons for differences between the materials likely reflect changes in the chemistry of the particles and the material, which are also responsible for other properties which have been altered, to give such faster setting times and improved strength.

According to the manufacturer, these new materials use smaller glass particles to alter the early physical properties [12,13]. A useful potential clinical application for more acid resistant GIC materials would be as a bulk restorative material for cervical restorations (for root surface caries), and as well as for open sandwich restorations, where the GIC is overlaid with a posterior resin composite material, as in part two of the present study. To inform such applications, further research should be conducted to compare the clinical performance of these novel GIC materials versus older traditional GIC materials in patients with acidic oral environments. In conjunction with this, laboratory studies should measure not only mass loss and surface degradation, but other forms of deterioration, and assess any protective effects on adjacent tooth structure. Methods for such assessments are well established in the dental materials literature [18,19,20,21,22,23,24,25,26,27,28,29,30].

In the present study, F9X showed relatively high acid solubility. F9X has a higher fluoride release than F9F, as a result of its more reactive glass particles [31]. This could explain its increased solubility in citric and phosphoric acids. Conversely, EFF was less soluble than F9X and F7 discs in citric acid, which likely reflects the new hybrid glass used in this material to give it additional strength. FB is marketed as being a soluble conventional set GIC material that is more resistant to acidic oral environments [13], and this claim is supported by the current study.

The present results also reveal how exposure to water alone can influence some GIC materials. F7 gained approximately 2% in mass through water uptake at day 3, and this was sustained until day 7. The manufacturer’s directions for use for this particular material point out that—unlike other GIC materials—no isolation is required, and the material can be placed in a moist field. With this product, high water tolerance is regarded as a clinical advantage for situations when tooth surface protection is needed, but moisture control is challenging. The higher water sorption could be due to a range of factors related to the composition of the material, which uses a strontium-based glass and is designed to give sustained high release of fluoride ions to give protection to adjacent tooth structure [31,32]. Reducing the level of carboxylic acids in the liquid component of the cement to enhance its flow characteristics could mean that less crosslinking occurs between the polymer chains as the material undergoes its setting reaction to form calcium and strontium polyacrylates. This would increase the volume of spaces in the cement, and thus allow greater sorption of water [33]. The observation that the mass gain stabilized over time is consistent with the known reactions of GIC materials in the days after the initial setting reaction, when the amount of bound water rises but then reaches a plateau [34,35].

In the present study, F9X underwent a median mass loss of around 1.5% after 1 week in water, which was significantly greater than F9 and other GIC materials. As already mentioned, F9X also had a relatively high solubility in acid, compared with other materials, so it is not surprising that this particular material showed some solubility in water. Water can diffuse from—as well as into—GIC, particularly as the cement undergoes maturation over 4–6 weeks [34]. It is likely that the highly reactive glass particles allowed greater diffusion of various ions from the set cement, to cause this mass loss.

The current international standard for GIC [36] specifies requirements and test methods for powder/liquid acid-base dental cements intended for permanent cementation, lining and restoration, but does not specify particular limits on solubility in water or in various acids. Clinically, intermittent exposure of dental restorations to a wide range of aqueous fluids can be expected, both from ingested foods and beverages, as well as from cariogenic dental plaque and gastric reflux. In the case of lactic acid, the present study used 0.1% for one week. For comparison, the level of lactic acid in fermented milk products is typically 0.6 to 1.2% [37]. Following an approach of estimating exposure as concentration multiplied by time, 0.1% lactic acid for one week equates to approximately 16.8 h in a fermented milk product with 1% lactic acid. For citric acid, typical levels in fresh lemon juice and in lemonade beverages are 45% and 5%, respectively [17]. Hence, 10% citric acid for 1 week corresponds to 37 h in lemon juice, or 2 weeks in lemonade. Finally, for phosphoric acid, typical levels are 37% in dental etching gels and 0.15% in cola drinks (food additive E338) [38]. Thus, 0.2% phosphoric acid for 7 days corresponds to 54 min with etching gel, and to 5 days and 6 h in cola drinks.

The present study provided some insight into the difference between the interface of a GIC with tooth structure, compared to a resin composite material. Conventional GIC materials can release fluoride ions into the adjacent tooth structure and into dental plaque fluid, promoting remineralization and giving cariostatic actions [39]. Calcium and phosphate ion release during this process results in some sacrificial loss of the GIC at the GIC–tooth interface [40], and this was witnessed by a color change and softening of the GIC of the sandwich restoration. Tooth structure changes were, nevertheless, more severe at the interface of the tooth and the resin composite restoration resin–tooth interface. This differential effect has been seen in previous studies [15].

The reasons for the differing effects of the acids used reflect their fundamental chemistry. Both phosphoric and citric acids have well documented erosive effects on dental structures. The pH of the solutions used was lower for phosphoric acid (pH 2–3) than for citric acid (pH 3.5–4.0) or lactic acid (pH 4–4.5). Nevertheless, citric acid has a larger equilibrium constant, with higher dissociation constants for its second and third protons, allowing increased exchange of protons [41,42]. This allows citric acid to perform better as a chelator, which explains the marked extent of GIC dissolution in citric acid. When considering lactic acid, only small mass and surface changes occurred. This can be explained by reactions with the GIC matrix, which releases polyacrylic acid ions that create a buffering action [30,43,44]. In this manner, conventional GIC materials can mitigate in part the effects of prolonged lactic acid exposure.

## 5. Conclusions

Overall, the present results show that new generation GIC materials have superior resistance to acid dissolution. No material could tolerate continuous exposure to a strong chelator challenge in the form of 10% citric acid. Given the common presence of citric acid in many beverages [17,45,46], further work is needed to provide even more resistance to this particular acid. Further changes in the design of GIC materials should also consider enhancing the level of protection of adjacent tooth structure. When considering placing GIC materials in open sandwich restorations, attention should be directed to reducing the frequency of dietary acids, and elevating the salivary pH to reduce erosive events.
